# The Roles of Endoplasmic Reticulum Overload Response Induced by HCV and NS4B Protein in Human Hepatocyte Viability and Virus Replication

**DOI:** 10.1371/journal.pone.0123190

**Published:** 2015-04-13

**Authors:** Lingbao Kong, Shanshan Li, Mingjie Huang, Ying Xiong, Qinghua Zhang, Li Ye, Jing Liu, Xiangdong Zhu, Ruina Sun, Yunli Guo

**Affiliations:** 1 College of Bioscience and Engineering, Jiangxi Agricultural University, Nanchang, Jiangxi, China; 2 Department of Plant Pathology & Microbiology, Iowa State University, Ames, Iowa, United States of America; 3 Jiangxi Province Center for Disease Control and Prevention, Nanchang, Jiangxi, China; 4 School of Public Health, Guangxi Medical University, Nanning, Guangxi, China; 5 Ministry of Education Laboratory of Combinatorial Biosynthesis and Drug Discovery, Wuhan University School of Pharmaceutical Science, Wuhan, Hubei, China; University of Montreal Hospital Research Center (CRCHUM), CANADA

## Abstract

Hepatitis C virus (HCV) replication is associated with endoplasmic reticulum (ER) and its infection triggers ER stress. In response to ER stress, ER overload response (EOR) can be activated, which involves the release of Ca^2+^ from ER, production of reactive oxygen species (ROS) and activation of nuclear factor κB (NF-κB). We have previously reported that HCV NS4B expression activates NF-κB via EOR-Ca^2+^-ROS pathway. Here, we showed that NS4B expression and HCV infection activated cancer-related NF-κB signaling pathway and induced the expression of cancer-related NF-κB target genes via EOR-Ca^2+^-ROS pathway. Moreover, we found that HCV-activated EOR-Ca^2+^-ROS pathway had profound effects on host cell viability and HCV replication. HCV infection induced human hepatocyte death by EOR-Ca^2+^-ROS pathway, whereas activation of EOR-Ca^2+^-ROS-NF-κB pathway increased the cell viability. Meanwhile, EOR-Ca^2+^-ROS-NF-κB pathway inhibited acute HCV replication, which could alleviate the detrimental effect of HCV on cell viability and enhance chronic HCV infection. Together, our findings provide new insights into the functions of EOR-Ca^2+^-ROS-NF-κB pathway in natural HCV replication and pathogenesis.

## Introduction

ER is a cellular organelle that controls several critical aspects of cellular processes such as cellular protein folding and post-translational modifications. Increasing evidence indicates that virus infection often disturbs ER homeostasis and leads to ER stress response, which has profound effects on virus replication and pathogenesis [1,2]. ER stress activates several intracellular signal pathways including unfolded protein response (UPR) [3] and ER overload response (EOR). UPR is initiated by phosphorylation of protein kinase R (PKR)-like ER kinase (PERK), cleavage of inositol-requiring enzyme 1 (IRE1) and proteolysis of activating transcription factor 6 (ATF6), which function to attenuate ER stress by inhibiting translation, degrading mRNA and increasing ER folding capacity, respectivley [2]. Unlike UPR, EOR pathway is characterized by release of Ca^2+^ from ER lumen to stimulate reactive oxygen species (ROS) production, which then activates NF-κB, namely EOR-Ca^2+^-ROS-NF-κB pathway [1,4]. NF-κB is a sequence specific transcription factor that regulates expression of many cellular genes such as genes involved in cancer cell survival (Mcl-1), proliferation (C-myc, Cyclin D1), and invasion (matrix metalloproteinase MMP-9), which play important roles in carcinogenesis [2,5,6,7]. In vivo studies using rodent models of liver disease and cell-targeted perturbation of NF-κB activity revealed that NF-κB has complex functions in liver survival and diseases such as hepatocellular carcinoma (HCC) [8]. However, the precise role of EOR-mediated NF-κB in HCV-caused liver diseases remains unknown.

HCV is a positive-strand RNA virus of the family *Flaviviridae*. Its open reading frame (ORF) encodes at least 10 viral proteins with the following order: NH_2_-C-E1-E2-p7-NS2-NS3-NS4A-NS4B-NS5A-NS5B-COOH [9]. Accumulating evidence indicates that NF-κB is involved in HCV replication and pathogenesis. NF-κB has been reported to be modulated by expression of full-length HCV ORF, Core, NS3, NS4B and NS5A [10,11,12,13]. In addition, HCV infection in human hepatocytes has been shown to activate NF-κB, which exhibits multiple functions. NF-κB activated by HCV infection via Toll like receptor-3 (TLR3) leads to production of chemokines and inflammatory cytokines such as RANTES, MIP-1β, MIP-1α, IL-6, IP-10 and TNF-α [14]. HCV-activated NF-κB also inhibits HCV replication and promotes T-helper 17 responses, although the underlying mechanisms remain elusive [15,16]. Furthermore, NF-κB is activated in the liver tissues from HCV-infected HCC patients, suggesting that NF-κB may be involved in HCC development [17]. Another report shows that NF-κB activity is inhibited in liver tissues from human end-stage HCV liver disease, suggesting that blunted NF-κB activation may be involved in more severe disease progression [18].

HCV NS4B is a 27 kDa ER membrane-associated protein that plays important roles in HCV replication and pathogenesis [19]. It induces alteration of ER membrane and formation of a ‘membranous web’ structure, which provides a platform for HCV replication complex [20]. We have previously reported that both transient and stable expression of HCV NS4B triggers ER stress and activates EOR-Ca^2+^-ROS-NF-κB pathway [1]. However, little is known about the functions of ER stress-activated NF-κB pathway in HCV replication and pathogenesis. Here, we found that NS4B- and HCV-activated EOR-Ca^2+^-ROS-NF-κB pathway induced cancer-related gene expression in human hepatocytes, implying that this pathway could be involved in carcinogenesis. Moreover, HCV-activated EOR-Ca^2+^-ROS pathway reduced human hepatocyte viability. As a feedback mechanism, the EOR-Ca^2+^-ROS-NF-κB pathway increased cell viability and inhibited acute HCV replication, which could enhance chronic HCV infection.

## Materials and Methods

### Cells and plasmids

Huh7.5.1 cells were kindly provided by Dr. Francis V. Chisari (The Scripps Research Institute, USA). Primary human hepatocytes (PHH) were purchased from ScienCell Research Laboratories (CA, USA). Huh-7 cells that stably express HCV NS4B and Huh-7 cells stably transfected with empty vector have been previously constructed [10]. Plasmid pJFH1 that contains the full-length HCV genotype 2a JFH1 strain cDNA was kindly provided by Dr. Takaji Wakita (National Institute of Infectious Diseases, Japan). Huh7.5.1 cells were maintained at 37°C in Dulbecco’s modified Eagle’s medium (DMEM) (Gibco-BRL, USA), supplemented with 10% heat-inactivated fetal bovine serum (Hyclone, USA) in a 5% CO_2_/95% air humidified atmosphere. Huh-7 cells that stably express NS4B or stably transfected with empty vector were maintained in a similar medium with 200 μg/ml G418. PHH cells were maintained in Hepatocyte Medium according to the manufacturer’s protocol (ScienCell Research Laboratories, USA).

### Quantitative Real-time RT–PCR

Real-Time RT-PCR was performed as described previously [21,22]. RNA quantification of JFH1, NS4B, C-myc, Mcl-1, Cyclin D1, MMP-9 and GAPDH were performed with SYBR green and gene specific primers (S1 Table). GAPDH was used as an endogenous reference to normalize the quantities of target RNA.

### RNA interference

To silence NS4B, RNA interference experiments were performed on Huh-7 cells that stably express NS4B using NS4B siRNA (Biomics, USA) and Lipofectamine RNAiMAX Reagent according to the manufacturer’s protocol (Invitrogen, USA). Knockdown of NS4B was confirmed by Real-Time RT-PCR and Western blot. Scrambled siRNA was used as a negative control.

### Western blot analysis

Total cell extracts and nuclear extracts were prepared as described [10]. Protein samples were separated by SDS-PAGE and transferred to nitrocellulose membranes. The blots were probed with primary antibodies against HCV Core, NS4B, phospho-IκBα (Tyr-42), p50, C-myc, Mcl-1, Cyclin D1, MMP-9, YY1 and actin. Next, horseradish peroxidase-labeled IgG as secondary antibodies were applied onto the blots. The specific proteins were visualized by using the ECL Chemiluminescence Detection Kit (Amersham Biosciences, USA).

### Luciferase assay

Cells on 24-well or 96-well tissue culture plates were transfected with pNF-κB-Luc and pRL-CMV as indicated. Two days after transfection, luciferase activity was measured by dual-luciferase assay system according to the protocol recommended by the manufacturer (Promega, USA). The firefly luciferase activity was normalized to the Renilla luciferase activity.

### Virus production and infection

Plasmid pJFH1, containing the full-length cDNA of the HCV genotype 2a JFH1 isolate, was used to generate infectious HCV particles in Huh7.5.1 cell culture as described previously [3]. In vitro transcribed JFH1 RNA was digested with DNase before transfecting into Huh7.5.1 cells. HCV particles were collected from cell culture media at day 21 posttransfection, which was centrifuged, passed through a 0.22 μm filter, and concentrated by ultrafiltration using a 500-kilodalton cut-off membrane. Virus was quantitated using HCV RNA qPCR diagnostic kit as described previously [23]. The viral RNA titer was expressed as IU/ml, determined by comparing to standards according to the manufacturer’s protocol (KHB Company, China). Aliquots were stored at -80°C prior to use. Human hepatocytes were infected with JFH-1 at a virus titer (IU/cell) as indicated.

### Cell viability assays

Cell viability was assessed using Cell Titre-Glo assay (Promega, USA) and Water-Soluble Tetrazolium (WST)-1 assay (Beyotime, China) according to the manufacturer’s protocols (Promega and Beyotime). All experiments were conducted in triplicate.

### Indirect Immunofluorescence

Infected Huh-7 cells were grown on a glass slide for 24 h and fixed in acetone-methanol (1:1) for 10 min at -20°C. Cells were blocked, and then incubated with mouse anti-Core antibodies. After washing with PBS, cells were incubated with Alexa FluorR anti-mouse secondary antibody (Invitrogen, USA). Cell nuclei were stained by DAPI (Sigma-Aldrich, USA). Stained samples were then examined with a Leica TCS SPII confocal microscope (Leica Microsystems, Germany).

### Other reagents

Ca^2+^ signaling inhibitors (Ca^2+^ chelator TMB-8, ER calcium channel blocker Ruthenium red, mitochondrial Ca^2+^ uniporter inhibitor Ryanodine) and antioxidant reagent, N-acetyl L-cysteine (NAC) were purchased from Sigma-Aldrich. NF-κB inhibitor, SN50 was purchased from Enzo Biochem, Inc. Antibodies against phospho-IκBα (Tyr-42), p50, actin and YY1 were purchased from Santa Cruz Biotechnology. Antibodies against Core and NS4B were purchased from Abcam. Antibodies against C-myc, Mcl-1, Cyclin D1 and MMP-9 and HRP-linked secondary antibody were purchased from Cell Signaling Technology.

### Statistical analysis

The method of relative quantification was used for analysis of the results of real-time RT-PCR, cell viability and luciferase assays. Statistical analysis was performed using the Statistical Package Social Sciences (SPSS) program version 11.5 by one-way analysis of variance (ANOVA) and significant differences among groups were determined by Least Significant Difference (LSD). The accepted level of statistical significance was p < 0.05.

## Results

### NS4B activates cancer-related NF-κB signaling pathway via EOR-Ca^2+^-ROS pathway in human hepatocytes

**Fig 1:**
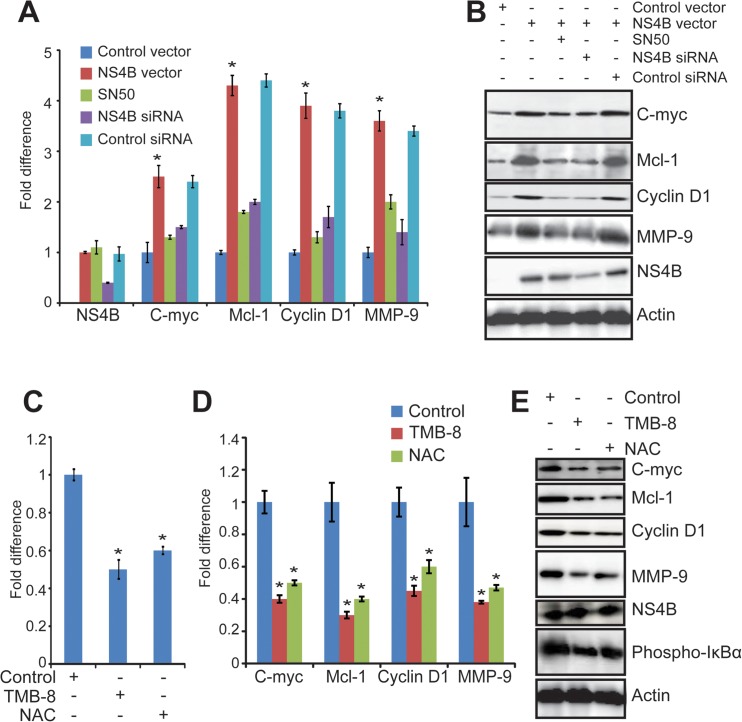
NS4B activates cancer-related NF-κB signaling pathway via EOR in human hepatocytes. Huh-7 cells stably expressing NS4B and control cells were treated with NF-κB inhibitor SN50 (40 μM) for 4 h or untreated as indicated. To show this effect is specific to NS4B, Huh-7 cells that stably expressed NS4B were treated with NS4B siRNA or control siRNA at a final concentration of 25 nM. (A). Real-time RT-PCR analysis of NS4B, C-myc, Mcl-1, Cyclin D1, and MMP-9. GAPDH acts as internal control. Values are means ± SD (n = 3). * P < 0.05. (B). Western blot analysis of the protein levels of C-myc, Mcl-1, Cyclin D1, MMP-9, and NS4B. Actin protein was used as internal controls. (C) and (D). Huh-7 cells stably expressing NS4B were co-transfected with plasmids pNF-κB-Luc and pRL-CMV, and treated with TMB-8 (100 μM) for 4 h and NAC (30 mM) for 8 h as indicated. At 48 h posttransfection, cells were subjected to luciferase assay for NF-κB activation (C) and real-time RT-PCR for analyzing transcripts of C-myc, Cyclin D1, Mcl-1 and MMP-9 (D). Values are means ± SD (n = 3). * P < 0.05. E. Huh-7 cells stably expressing NS4B were treated with TMB-8 (100 μM) for 4 h and NAC (30 mM) for 8 h as indicated. The protein levels of C-myc, Mcl-1, Cyclin D1, HCV NS4B, MMP-9 and phospho-IκBα were analyzed by western blot with indicated antibodies. Phospho-IκBα was probed to confirm NF-κB activation.

We have previously reported that expression of HCV NS4B in Huh-7 cells activates NF-κB [1]; however, it remains unknown about which NF-κB downstream genes are induced by NS4B. As HCV infection leads to HCC, the effects of NS4B on NF-κB target genes related to cancer were analyzed by real time RT-PCR and Western blot. As shown in Fig 1A, stable expression of NS4B in Huh-7 cells significantly increased the transcription of C-myc, Mcl-1, Cyclin D1 and MMP-9 (p<0.05). This effect is specific to NS4B as knockdown of NS4B by siRNA significantly decreased NS4B-induced transcription of C-myc, Mcl-1, Cyclin D1 and MMP-9 in Huh-7 cells. Moreover, NF-κB inhibitor, SN50, suppressed the transcription of C-myc, Mcl-1, Cyclin D1 and MMP-9 in Huh-7 cells that stably expressed NS4B, indicating that NS4B induced the transcription of C-myc, Mcl-1, Cyclin D1 and MMP-9 by NF-κB (Fig 1A). Consistent with the above results, the protein levels of C-myc, Mcl-1, Cyclin D1 and MMP-9 were also increased by NS4B expression while NS4B siRNA and SN50 treatments significantly suppressed NS4B-induced expression of C-myc, Mcl-1, Cyclin D1 and MMP-9 in Huh-7 cells (Fig 1B). These results indicate that NS4B induces the expression of cancer-related genes (C-myc, Mcl-1, Cyclin D1 and MMP-9) via NF-κB in human hepatoma cells.

Since our previous work has shown that NS4B expression activates NF-κB by EOR via Ca^2+^ signaling and ROS production [1], we next examined whether these signaling pathways are involved in NS4B-induced expression of NF-κB cancer-related target genes. Huh-7 cells that stably expressed NS4B were treated with Ca^2+^ chelator TMB-8 or antioxidant reagent NAC, which inhibit Ca^2+^ signaling and ROS production, respectively. Our results showed that TMB-8 and NAC treatments significantly decreased NF-κB activity (Fig 1C, p<0.05) as well as transcription of NF-κB target genes, C-myc, Mcl-1, Cyclin D1 and MMP-9 (Fig 1D, p<0.05). Further studies showed that both TMB-8 and NAC decreased the protein levels of C-myc, Mcl-1, Cyclin D1, and MMP-9 as well as IκBα phosphorylation (Phospho-IκBα) in Huh-7 cells that stably expressed NS4B (Fig 1E). Collectively, these results indicate that expression of NS4B in human hepatocytes induces cancer-related NF-κB target genes including C-myc, Mcl-1, Cyclin D1 and MMP-9 via EOR-Ca^2+^-ROS-NF-κB pathway.

### Stable NS4B expression promotes human hepatocyte viability by EOR-Ca^2+^-ROS-NF-κB pathway

**Fig 2:**
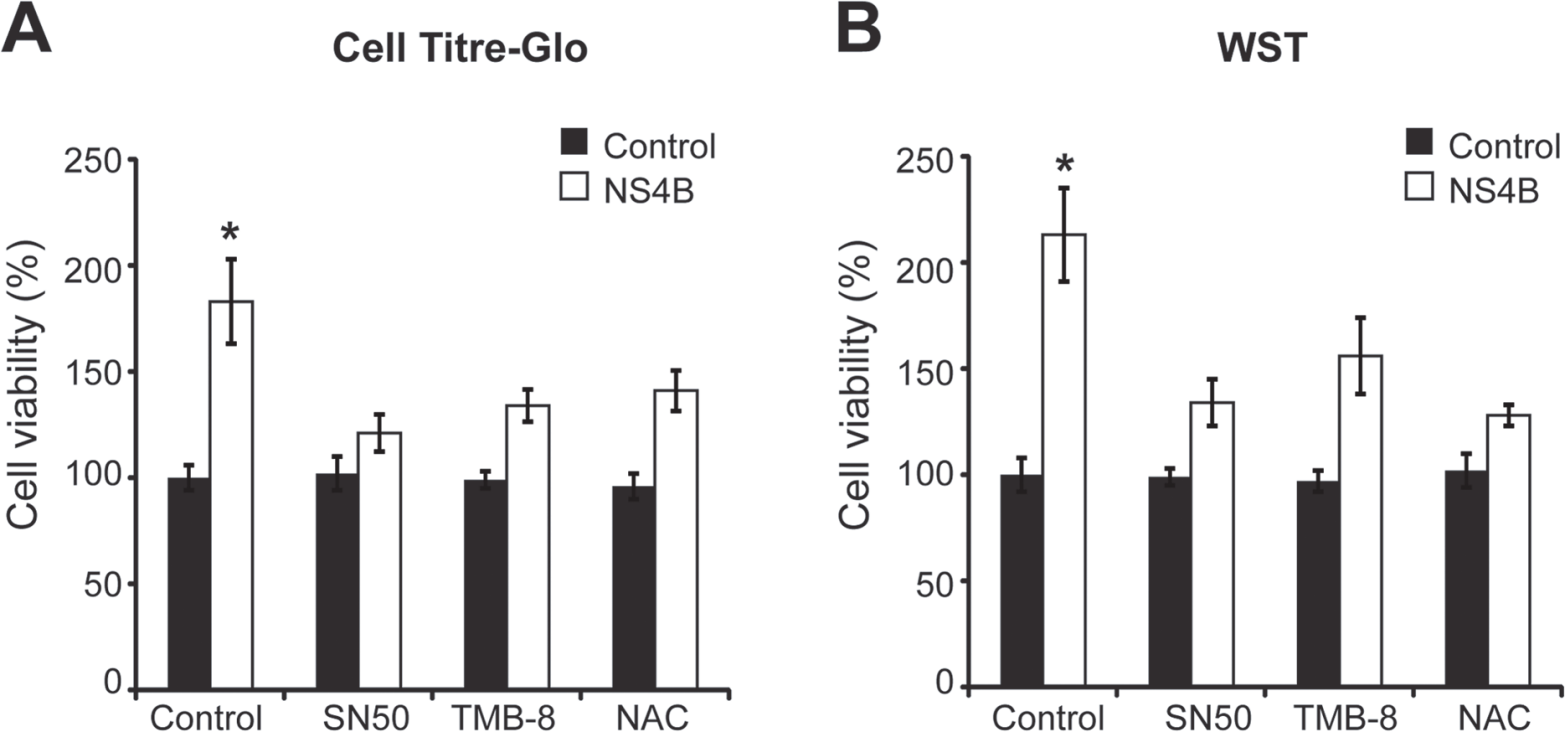
The effect of NS4B on human hepatocyte viability via EOR. Huh7 cells stably expressing NS4B or control cells were plated on 24-well plates and treated with SN50 (40 μM) for 4 h, NAC (30 mM) for 8 h and TMB-8 (100 μM) for 4 h as indicated. After 48 h, cell viability was assessed using Cell Titre-Glo (A) and WST (B) assays. Values are means ± SD (n = 3). * P < 0.05.

As NS4B induced the expression of four cancer-related genes by EOR-Ca^2+^-ROS-NF-κB pathway in human hepatocytes, the effects of NS4B on human hepatocyte viability were investigated by Cell Titre-Glo (Fig 2A) and WST assays (Fig 2B). Stable expression of NS4B in Huh-7 cells significantly increased cell viability (p<0.05) when compared to the control cells. SN50, TMB-8 and NAC treatments significantly decreased viability of Huh-7 cells with stable NS4B expression (Fig 2). However, SN50, TMB-8 and NAC treatments had no significant effects on viability of the control cells (Fig 2), suggesting that stable NS4B expression in human hepatocyte predominantly increases cell viability by EOR-Ca^2+^-ROS-NF-κB pathway.

### HCV infection activates NF-κB signaling pathway related to cancer in human hepatocytes via EOR-Ca^2+^-ROS pathway

**Fig 3:**
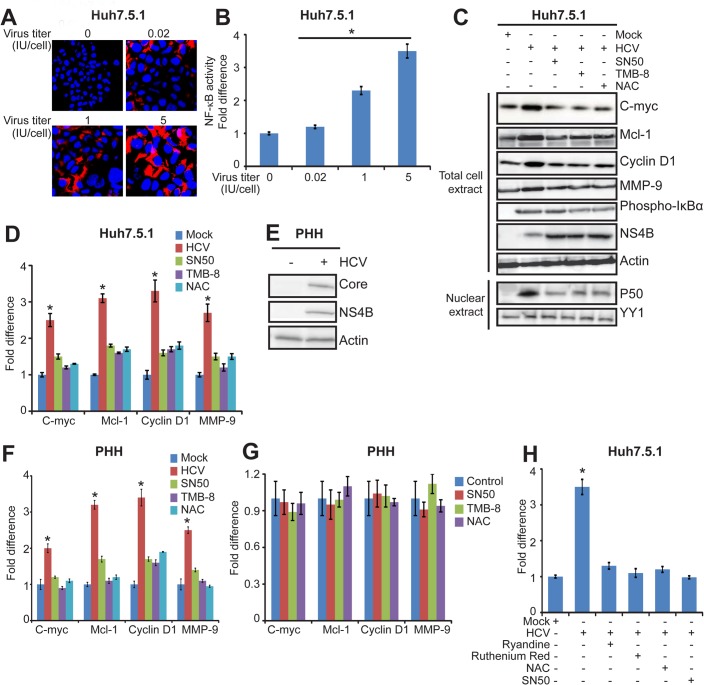
Activation of cancer-related NF-κB signaling pathway by HCV via EOR in human hepatoma cells. (A). Huh7.5.1 cells were infected with JFH1 at a virus titer (IU/cell) of 0, 0.02, 1 and 5. At 48 h postinfection, cells were subjected to indirect immunofluorescece with mouse anti-Core antibody and Alexa FluorR 555 anti-mouse secondary antibody. Nuclei were stained with DAPI. (B). Huh7.5.1 cells were infected with JFH1 at a virus titer (IU/cell) of 0, 0.02, 1 and 5, and transfected with plasmids consisting of NF-κB-Luc and pRL-CMV. After 48 h, cells were subjected to luciferase assay for NF-κB activation. Values are means ± SD (n = 3). * P < 0.05. Scale bars represent 50 μm. (C) and (D). Huh7.5.1 cells were infected with JFH1 at a virus titer (IU/cell) of 5, and treated with SN50 (40 μM) for 4 h, TMB-8 (100 μM) for 4 h and NAC (30 mM) for 8 h as indicated. (C). Western blot analysis of protein levels of C-myc, Mcl-1, Cyclin D1, MMP-9, phospho-IκBα, NS4B and actin in cells at 48 h posttransfection. Actin protein bands act as internal control. p50 protein accumulation in the nuclear extracts was also analyzed by western blot. YY1 acts as a nuclear-specific control. (D). Real-time RT-PCR analysis of C-myc, Mcl-1, Cyclin D1 and MMP-9. GAPDH act as internal control. Values are means ± SD (n = 3). * P < 0.05. (E). Primary human hepatocytes in 24-well plate were infected with JFH1 at a virus titer (IU/cell) of 1. Mock-infected primary human hepatocytes were used as controls. At 48 h postinfection, Core, NS4B and actin proteins were determined by Western blot. (F). Primary human hepatocytes in 96-well plates were infected with JFH1 at a virus titer (IU/cell) of 1, and treated with SN50 (40 μM) for 4 h, TMB-8 (100 μM) for 4 h and NAC (30 mM) for 8 h as indicated. At 48 h postinfection, transcripts of C-myc, Mcl-1, Cyclin D1 and MMP-9 in cells were analyzed by real-time RT-PCR. GAPDH acts as internal control. Values are means ± SD (n = 3). * P < 0.05. (G). Mock-infected primary human hepatocytes in 96-well plates and treated with SN50 (40 μM) for 4 h, TMB-8 (100 μM) for 4 h and NAC (30 mM) for 8 h as indicated. At 48 h postinfection, transcripts of C-myc, Mcl-1, Cyclin D1 and MMP-9 in cells were analyzed by real-time RT-PCR. GAPDH acts as internal control. Values are means ± SD (n = 3). * P < 0.05. (H). Huh7.5.1 cells were infected with JFH1 at a virus titer (IU/cell) of 5, transfected with plasmids consisting of NF-κB-Luc and pRL-CMV, and treated with SN50 (40 μM) for 4 h, Ryanodine (100 nM) for 4 h, Ruthenium red (50 μM) for 4 h and NAC (30 mM) for 8 h as indicated. At 48 h posttranfection, cells were subjected to luciferase assay. Values are means ± SD (n = 3). * P < 0.05.

Having established that NS4B activates NF-κB target genes related to cancer, the next question is whether HCV infection activates NF-κB and its downstream target genes. Huh7.5.1 cells were infected with JFH1 at different titers (Fig 3A) and analyzed for the activation of NF-κB signaling pathway related to cancer. HCV induced NF-κB activation in Huh7.5.1 cells and this activation was viral titer-dependent (Fig 3B, p<0.05). Moreover, we found that the expression of NF-κB target genes including C-myc, Mcl-1, Cyclin D1 and MMP-9 was induced in HCV-infected Huh7.5.1 cells and NF-κB inhibitor SN50 suppressed the expression of these NF-κB target genes (Fig 3C and 3D).

As HCV JFH1 has been reported to infect primary human hepatocytes [10], we also transfected HCV JFH1 into primary human hepatocytes and examined the transcription of NF-κB target genes C-myc, Mcl-1, Cyclin D1 and MMP-9. In JFH1-infected primary human hepatocytes, we detected HCV RNA (S1 Fig) and HCV Core and NS4B proteins (Fig 3E), indicating that HCV undergoes effective replication in primary human hepatocytes. We observed that HCV infection significantly enhanced the transcription of NF-κB target genes and SN50 suppressed their transcription (Fig 3F). These data demonstrate that HCV infection activates NF-κB signaling pathway related to cancer in human hepatocytes.

To investigate whether HCV infection activates NF-κB by ER stress via Ca^2+^ signaling and ROS production, HCV-infected cells were treated with Ca^2+^ chelator TMB-8 or antioxidant reagents NAC. TMB-8 and NAC treatments significantly suppressed the expression of NF-κB target genes, C-myc, Mcl-1, Cyclin D1 and MMP-9 in both HCV-infected Huh7.5.1 cells (Fig 3C and 3D) and HCV-infected primary human hepatocytes (PHH) (Fig 3F). In contrast, these treatments had no significant effect on NF-κB target genes in mock cells (Fig 3G). These results indicate that HCV infection specifically activates NF-κB via EOR-Ca^2+^-ROS pathway, which is consistent with data reported by Saddiqui and colleagues that HCV subgenomic replicon activates NF-κB via EOR and calcium chelator treatment inhibits subgenomic HCV replicon-induced NF-κB activity [11].

As EOR can be caused by release of Ca^2+^ from ER into cytosol, which is then transported into mitochondria to stimulate ROS production [24], we studied whether HCV activated NF-κB by these temporal events via ER and mitochondria. To block the calcium release from ER, we treated HCV-infected cells with Ryanodine, an ER calcium channel blocker, and found that Ryanodine treatment significantly reduced NF-κB activity (Fig 3H). To inhibit mitochondrial calcium uptake, we treated HCV-infected cells with Ruthenium Red, an inhibitor of calcium influx into mitochondria, and found that it reduced NF-κB activity to the same level with NAC and SN50 treatments (Fig 3H). These data suggest that both ER and mitochondria contribute to disturbance in calcium signaling in HCV-infected cells, which leads to the generation of ROS and activation of NF-κB as well as the expression of cancer-related genes.

### HCV infection decreases human hepatocyte viability by EOR-Ca^2+^-ROS

**Fig 4:**
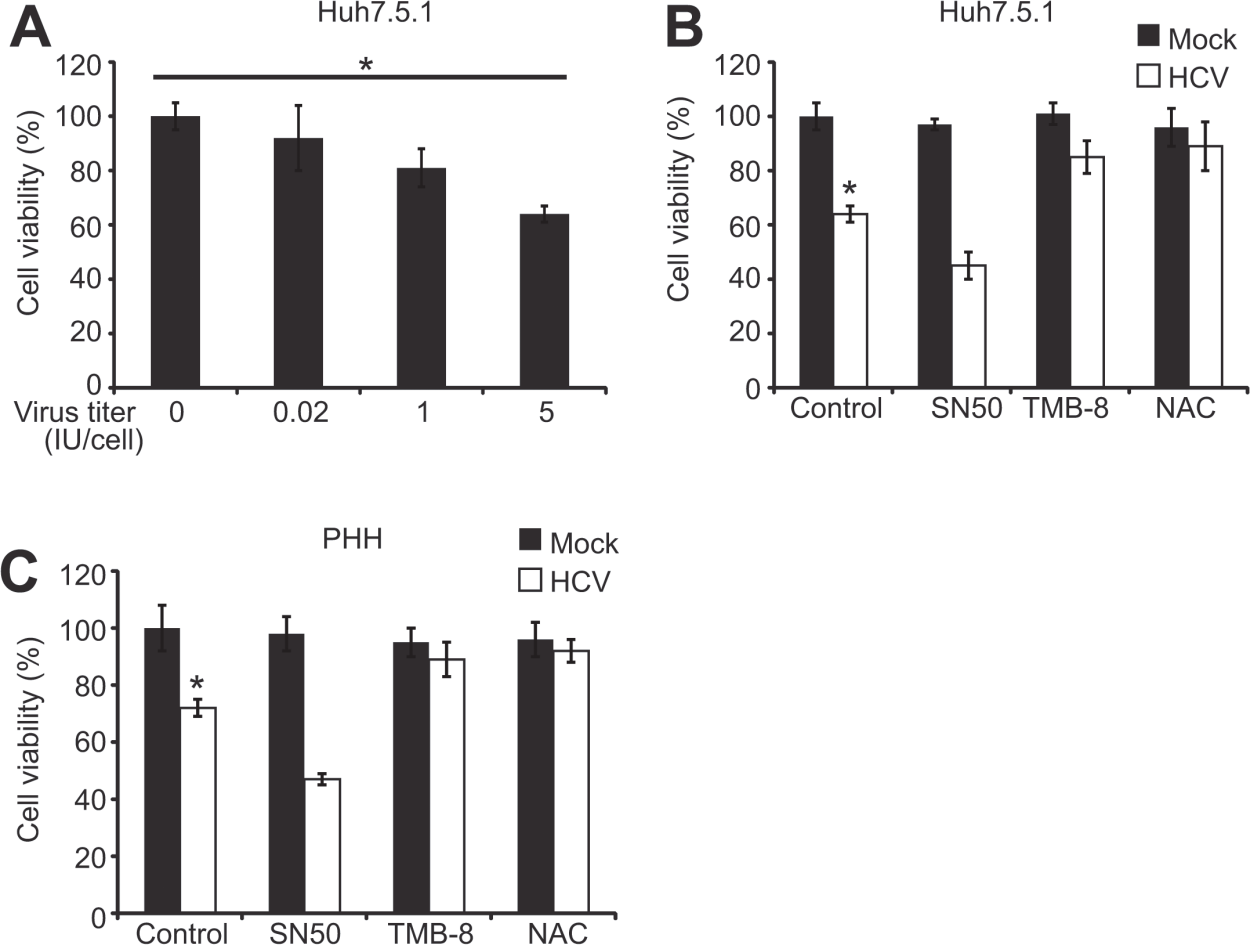
The effect of HCV on human hepatocyte viability via EOR. (A). Huh7.5.1 cells were infected with JFH1 at a virus titer (IU/cell) of 0, 0.02, 1 and 5. At 48 h postinfection, cell viability was assessed by using Cell Titre-Glo assay. Values are means ± SD (n = 3). * P < 0.05. (B). Huh7.5.1 cells were infected with JFH1 at a virus titer (IU/cell) of 5 and treated with TMB-8 (100 μM) for 4 h, NAC (30 mM) for 8 h and SN50 (40 μM) for 4 h as indicated. Mock-infected Huh7.5.1 cells were used as controls. At 48 h postinfection, cell viability was assessed by using Cell Titre-Glo assay. Values are means ± SD (n = 3). * P < 0.05. (C). Primary human hepatocytes in 96-well plates were infected with JFH1 at a virus titer (IU/cell) of 1 and treated with TMB-8 (100 μM) for 4 h, NAC (30 mM) for 8 h and SN50 (40 μM) for 4 h as indicated. Mock-infected primary human hepatocytes were used as controls. At 48 h postinfectiom, cell viability was assessed using Cell Titre-Glo assay. Values are means ± SD (n = 3). * P < 0.05.

Next, we investigated the effect of HCV on cell viability using Cell Titre-Glo and WST assays. In contrast to stablely expressed NS4B, Huh7.5.1 cell viability was significantly decreased with increasing titers of HCV infection (Fig 4A). Moreover, SN50 further decreased HCV-induced cell viability, whereas TMB-8 and NAC enhanced cell viability (Fig 4B). However, these treatments had no effect on cell viability of mock-infected cells (Fig 4B), indicating that HCV-activated EOR-Ca^2+^-ROS and EOR-Ca^2+^-ROS-NF-κB pathways have opposite effects on viability of HCV-infected cells. As we stably expressed NS4B in Huh-7 cells and infected Huh7.5.1 cells with JFH1, the discrepancy between stably expressed NS4B and JFH1 on cell viability could be caused by different cell lines used. To explore this possibility, we transiently transfected NS4B in Huh-7 and Huh7.5.1 cells and found that similar to JFH1 infection, NS4B transient expression reduced viability of both Huh-7 and Huh7.5.1 cells (S2 Fig, P<0.05), which is consistent with a recent report that transient expression of NS4B induces apoptosis in Huh-7 and 293T cells [25]. Moreover, treatment with SN50 further reduced cell viability but TMB-8 and NAC enhanced cell viability in NS4B transfected cells (S2 Fig, P<0.05), indicating that transiently expressed NS4B reduces cell viability via EOR-Ca^2+^-ROS pathway irrespective of cell lines.

We also examined the effect of HCV infection on primary human hepatocytes viability. As shown in Fig 4C, HCV infection significantly reduced PHH cell viability similar to Huh7.5.1. Moreover, SN50 treatment further reduced cell viability but TMB-8 and NAC treatments enhanced cell viability (Fig 4C). Like Huh7.5.1 cells, these treatments had no effect on cell viability of mock-infected PHH cells (Fig 4C). Together, these results indicate that HCV infection in human hepatocytes predominantly induces cell death by EOR-Ca^2+^-ROS although its downstream NF-κB inhibits cell death.

### Inhibition of EOR-Ca^2+^-ROS-NF-κB pathway activates HCV replication

**Fig 5:**
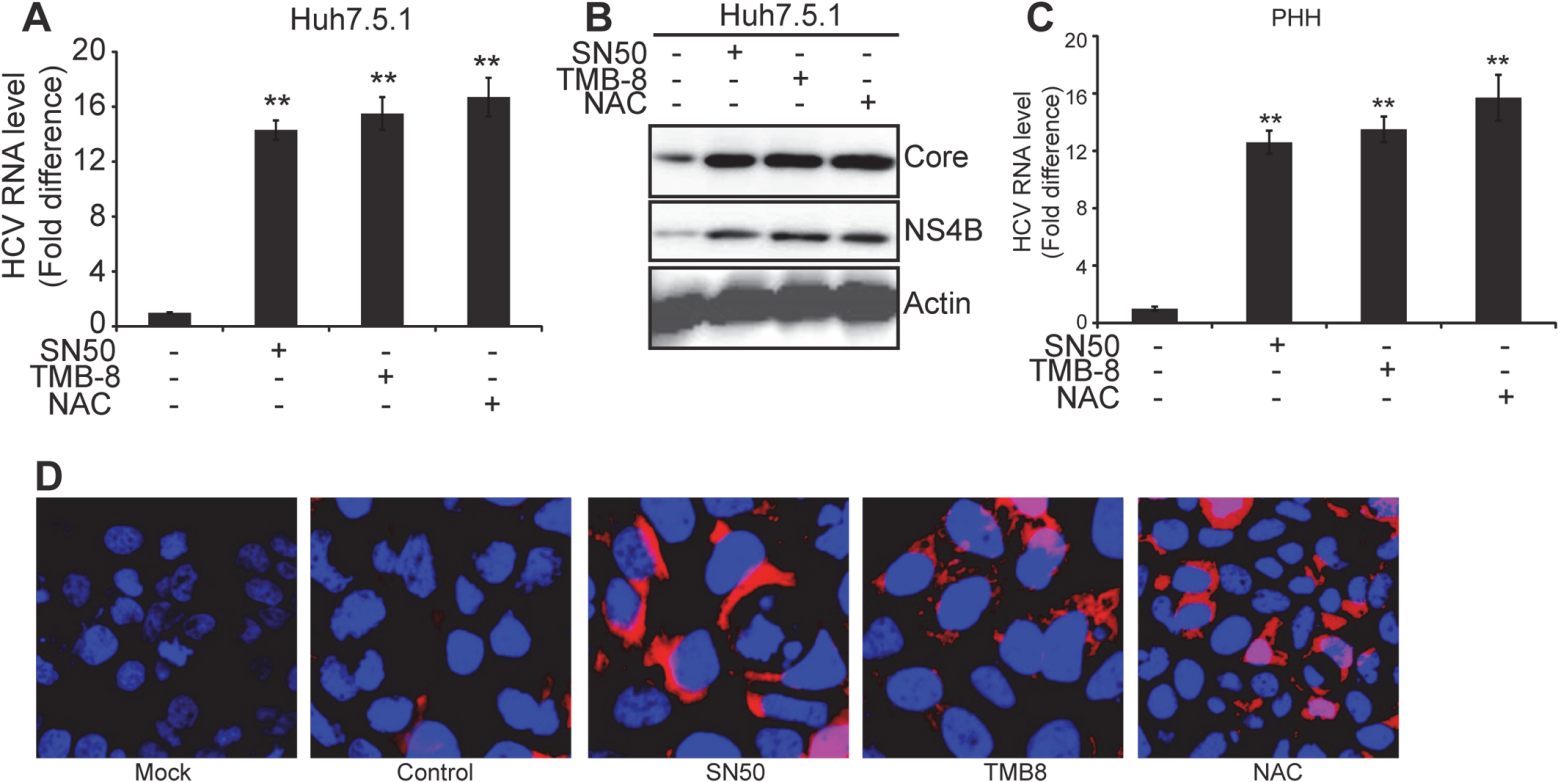
Inhibition NF-κB facilitates HCV replication in human hepatocytes. (A) and (B). Huh7.5.1 cells were infected with JFH1 at a virus titer (IU/cell) of 0.02 and treated with TMB-8 (100 μM) for 4 h, NAC (30 mM) for 8 h and SN50 (40 μM) for 4 h or untreated as indicated. At 72 h postinfection, cells were subjected to real-time RT-PCR for analyzing intracellular HCV RNA levels (A) and Western blot for analyzing intracellular HCV Core and NS4B protein levels (B). ** P < 0.01. (C). Primary human hepatocytes were infected with JFH1 at a virus titer (IU/cell) of 0.02 and treated with TMB-8 (100 μM) for 4 h, NAC (30 mM) for 8 h and SN50 (40 μM) for 4 h or untreated as indicated. At 72 h postinfection, cells were subjected to real-time RT-PCR for analyzing intracellular HCV RNA levels. GAPDH acts as internal control. Values are means ± SD (n = 3). ** P < 0.01. (D). Huh7.5.1 cells were infected with JFH1 at a virus titer (IU/cell) of 0.02 and treated with TMB-8 (100 μM) for 4 h, NAC (30 mM) for 8 h and SN50 (40 μM) for 4 h or untreated as indicated. At 72 h postinfection, cells were subjected to indirect immunofluorescence with mouse anti-Core antibody and Alexa FluorR 555 anti-mouse secondary antibody. Nuclei were stained with DAPI. The mock-infected cells were used as a control.

As shown in Fig 3C and S1 Fig, we found that NS4B expression and HCV RNA level in JFH1-infected cells were increased by treatment with SN50, TMB-8 and NAC, implying that EOR-Ca^2+^-ROS-NF-κB pathway affects HCV replication. To further investigate this effect, we quantitated HCV RNA levels in human hepatocytes infected with JFH1 HCV in the presence or absence of NF-κB inhibitor SN50. Our results showed that SN50 significantly increased HCV RNA levels in Huh7.5.1 cells (Fig 5A, P<0.01) and PHH cells (Fig 5C, P<0.01). Moreover, SN50 treatment increased the protein levels of Core and NS4B in Huh7.5.1 cells (Fig 5B), indicating that inhibition of NF-κB activates HCV replication. To examine whether NF-κB inhibits HCV replication via EOR-Ca^2+^-ROS pathway, we treated HCV-infected human hepatocytes with TMB-8 and NAC. Our data showed that like SN50, TMB-8 and NAC significantly increased JFH1 replication in human hepatocytes (Fig 5A, 5B and 5C). Collectively, these results indicate that inhibition of EOR-Ca^2+^-ROS-NF-κB pathway facilitates HCV replication.

To further visualize the effect of EOR-Ca^2+^-ROS-NF-κB on HCV replication, we treated JFH1-infected Huh7.5.1 cells with TMB-8, NAC andSN50 and monitored the intracellular expression of HCV proteins by indirect immunofluorescence at 72 h post-infection. As shown in Fig 5D, there was much more Core expression in TMB-8, NAC, and SN50 treated JFH1-infected Huh7.5.1 cells compared with untreated cells. All these evidence clearly indicate that EOR-Ca^2+^-ROS-NF-κB pathway regulates HCV replication.

## Discussion

HCV infection may lead to chronic hepatitis, liver cirrhosis and HCC, which cause a serious burden on global public health and hence prompt many efforts to elucidate HCV replication and pathogenesis [26]. ER stress has been reported to be triggered by many viruses and plays important roles in virus replication and pathogenesis [2]. Mounting evidence has shown that HCV infection or HCV protein expression activates ER stress in human hepatocytes [1,2]. However, little is known about the roles of ER stress in HCV replication and pathogenesis. In this study, we found that HCV and its protein NS4B induced the expression of cancer-related NF-κB target genes (C-myc, Mcl-1, Cyclin D1 and MMP-9) in both human hepatoma cells and primary human hepatocytes and this induction was mediated by ER stress response pathway, EOR-Ca^2+^-ROS, implying that ER stress response pathway may be involved in carcinogenesis. Moreover, we found that EOR-Ca^2+^-ROS-NF-κB pathway regulated human hepatocyte viability and HCV replication, which could contribute to chronic HCV infection and HCV pathogenesis. Together, our findings provide new insights into the roles of ER stress response pathway, EOR-Ca^2+^-ROS-NF-κB in natural HCV replication and pathogenesis.

NF-κB has been reported to be involved in inflammation and cancer [27] and was hypothesized to function in HCV-induced chronic hepatitis and HCC, respectively. It has been reported that NF-κB activates the expression of genes related to inflammation in human hepatocytes [14]. However, it remains unclear about the effects of NF-κB in the genes related to caner in human hepatocytes. In this study, we, for the first time, found that NS4B and HCV induced the expression of four cancer-related NF-κB target genes, C-myc, Mcl-1, Cyclin D1 and MMP-9 by the EOR-Ca^2+^-ROS-NF-κB pathway in both human hepatoma cells and primary human hepatocytes. These four genes have been reported to play important roles in HCC [28,29,30,31]. Over-expression of C-myc in hepatocytes has been shown to promote the onset of liver fibrosis [32]. Mcl-1 plays complex roles in HCC development through regulating cell apoptosis. During the early HCC stage, Mcl-1 could inhibit apoptosis to suppress HCC initiation; however, during the late stage, Mcl-1 inhibits apoptosis to facilitate HCC progression [33,34]. MMP-9 facilitates the motility and invasiveness of HepG2 cell [29]. Further studies showed that NS4B and HCV infection induced the expression of these four NF-κB target genes in human hepatocytes via Ca^2+^ signaling and ROS, suggesting that HCV could activate EOR-Ca^2+^-ROS-NF-κB pathway to cause HCC. Our data also indicated that HCV activated NF-κB might involve a temporal event via ER and mitochondria. Based on our results and published data [24], we proposed that HCV infection first triggered Ca^2+^ efflux from ER into cytoplasm and Ca^2+^ was then transported into mitochondria to stimulate ROS production. As a consequence, NF-κB and its downstream cancer-related genes were activated. NS5A has been reported to induce EOR-mediated NF-κB [35]. It is possible that NS5A could activate these four cancer-related genes and the activation effect of HCV could be the combined functions of NS4B and NS5A.

Our study provides evidence that HCV infection promotes cell death by EOR-Ca^2+^-ROS pathway. Interestingly, persistent expression of separate NS4B in human hepatoma cells promoted cell viability, consistent with its role in inducing NIH-3T3 transformation [36]. The difference between stably expressed NS4B and JFH1 infection on cell viability was not caused by cell lines as transiently expressed NS4B reduced the viability of both Huh-7 and Huh7.5.1 cells (S2 Fig). One possible explanation for this discrepancy is that other HCV proteins contribute to HCV-induced cell death. These proteins may include HCV Core, NS3, NS5A and NS5B as they induced cell death in mature dendritic cells [37]. Another possibility is related to different expression status of NS4B in these two systems (persistent expression of NS4B in NS4B stably transfected cells and transient expression of NS4B in JFH1-infected cells), since different NS4B expression states have distinct effects on cell viability. Our data clearly indicate that transiently expressed NS4B induced cell death whereas stably expressed NS4B promoted cell viability. Different expression states of HCV core protein have also been reported to have opposite effects on cell viability [38,39].

EOR-Ca^2+^-ROS and EOR-Ca^2+^-ROS-NF-κB pathways have distinct impacts on cell viability as shown by studies with specific inhibitors for Ca^2+^, ROS and NF-κB: EOR-Ca^2+^-ROS mediates cell death (Fig 4B and 4C; S2 Fig and S3 Fig), while EOR-Ca^2+^-ROS-NF-κB promotes cell viability (Figs 2, 4B and 4C; S2 Fig and S3 Fig). The functions of EOR-Ca^2+^-ROS in cell death could be due to oxidative stress and activation of apoptosis pathway as described previously [40, 41]. The cyto-protective effect of NF-κB could be mediated by its downstream targets that have anti-apoptotic effects or by cancer-related proteins, i.e. Mcl-1 [42]. Thus, ROS-activated NF-κB acts as a feedback mechanism to alleviate the detrimental effect of ROS on cell death. It is possible that the overall cell viability is reduced if the detrimental effect of ROS predominates the cyto-protective effect of NF-κB. Otherwise, cell viability is enhanced. HCV infection inhibits cell viability through Ca^2+^ signaling and ROS, and this inhibitory effect predominates the cyto-protective effect of the downstream NF-κB. As HCV infection can trigger ER stress and reduce cell viability in a dose-dependent manner (Fig 3A), it is probably that HCV infection triggers acute ER stress that overwhelms the overall capacity of ER. As an emergence mechanism, cells choose apoptosis to prevent more damage, which could explain HCV-induced liver damage.

NF-κB is regarded as a major regulator of the innate immune defense to virus infection since it activates a variety of antiviral genes. However, recent studies indicate that viruses have acquired the capability to reprogram this antiviral activity and exploit this factor for efficient replication [43,44,45]. To explore the roles of NF-κB in HCV replication, we studied its impact on JFH1 replication in both human hepatoma cells and primary human hepatocytes. Our results showed that NF-κB activation inhibited JFH1 replication in human hepatocytes, which is consistent with the recent reports that NF-κB activation inhibited the replication of JFH1 in human hepatoma cells [15,16]. However, in our study, NF-κB is activated by ER stress response pathway (EOR-Ca^2+^-ROS) not by protein kinase R (PKR) to inhibit HCV replication. As acute HCV infection always triggers cell apoptosis, the inhibitory effect of NF-κB on HCV replication could act as a feedback mechanism to enhance cell survival by attenuating HCV replication, which could favor chronic HCV infection.

Our results implied a mechanism by which ER stress regulates HCV replication and pathogenesis. HCV infection and NS4B expression in human hepatocytes trigger ER stress, which activates EOR-Ca^2+^-ROS pathway. The EOR-Ca^2+^-ROS pathway has two opposite effects on cell viability: EOR-stimulated ROS induces cell apoptosis; on the other hand, ROS could promote cell viability by activating NF-κB signaling pathway. HCV infection in human hepatocytes predominantly triggered hepatocyte death by activating Ca^2+^ signaling and stimulating ROS production, which could contribute to compensatory proliferative response of hepatocytes. Meanwhile, NF-κB could control HCV infection to attenuate its detrimental effect on cell viability, therefore enhancing chronic HCV infection. Therefore, our findings reveal a novel function of the EOR-Ca^2+^-ROS-NF-κB pathway in chronic HCV infection, providing new insights into natural HCV replication and pathogenesis.

## Supporting Information

S1 Fig Inhibition of EOR-Ca^2+^-ROS-NF-κB pathway increases HCV RNA level in JFH1-infected primary human hepatocytes. Primary human hepatocytes in 96-well plates were infected with JFH1 at a virus titer (IU/cell) of 1, and treated with SN50 (40 μM) for 4 h, TMB-8 (100 μM) for 4 h and NAC (30 mM) for 8 h as indicated. At 48 h postinfection, intracellular HCV RNA levels were determined by RT-PCR using JFH1-specific primers indicated in S1 Table, and the RT-PCR products were electrophoresed on 0.7% agarose gel. GAPDH acts as internal control.
(TIF)

S2 Fig Transient expression of NS4B induces cell death. Huh-7 (A) or Huh7.5.1 (B) cells in 24-well plates were transfected with 0.4 μg pcDNA3.1(−)NS4B or 0.4 μg pcDNA3.1 (−), and treated with SN50 (40 μM) for 4 h, TMB-8 (100 μM) for 4 h, and NAC (30 mM) for 8 h as indicated. At 48 h posttransfection, cell viability was assessed using Cell Titre-Glo assay. Values are means ± SD (n = 3). * P < 0.05.
(TIF)

S3 Fig The roles of EOR induced by HCV and NS4B in human hepatocyte viability. HCV and its protein NS4B induce the EOR-Ca^2+^-ROS pathway. Transient expression of NS4B and HCV infection induced cell death via Ca^2+^ signaling and ROS. Persistent expression of NS4B promoted human hepatocyte viability by Ca^2+^-ROS-activated NF-κB. SN50 specifically inhibits NF-κB, while TMB-8 and NAC specifically inhibit both EOR-Ca^2+^-ROS and EOR-Ca^2+^-ROS-NF-κB.
(TIF)

S1 Table Primers used in this study. (DOC)
