# Supplementary figures and images for: The Roles of Endoplasmic Reticulum Overload Response Induced by HCV and NS4B Protein in Human Hepatocyte Viability and Virus Replication
Source: PLoS One. 2015 Apr 13;10(4):e0123190. doi: 10.1371/journal.pone.0123190 (PMC4395406; doi:10.1371/journal.pone.0123190)

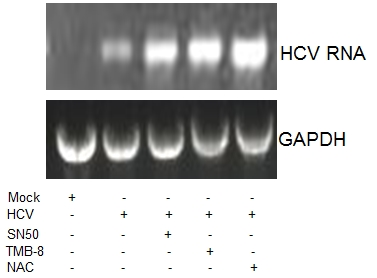

Supplement: S1 Fig — Primary human hepatocytes in 96-well plates were infected with JFH1 at a virus titer (IU/cell) of 1, and treated with SN50 (40 μM) for 4 h, TMB-8 (100 μM) for 4 h and NAC (30 mM) for 8 h as indicated. At 48 h postinfection, intracellular HCV RNA levels were determined by RT-PCR using JFH1-specific primers indicated in S1 Table, and the RT-PCR products were electrophoresed on 0.7% agarose gel. GAPDH acts as internal control. (TIF) [file pone.0123190.s001.tif]

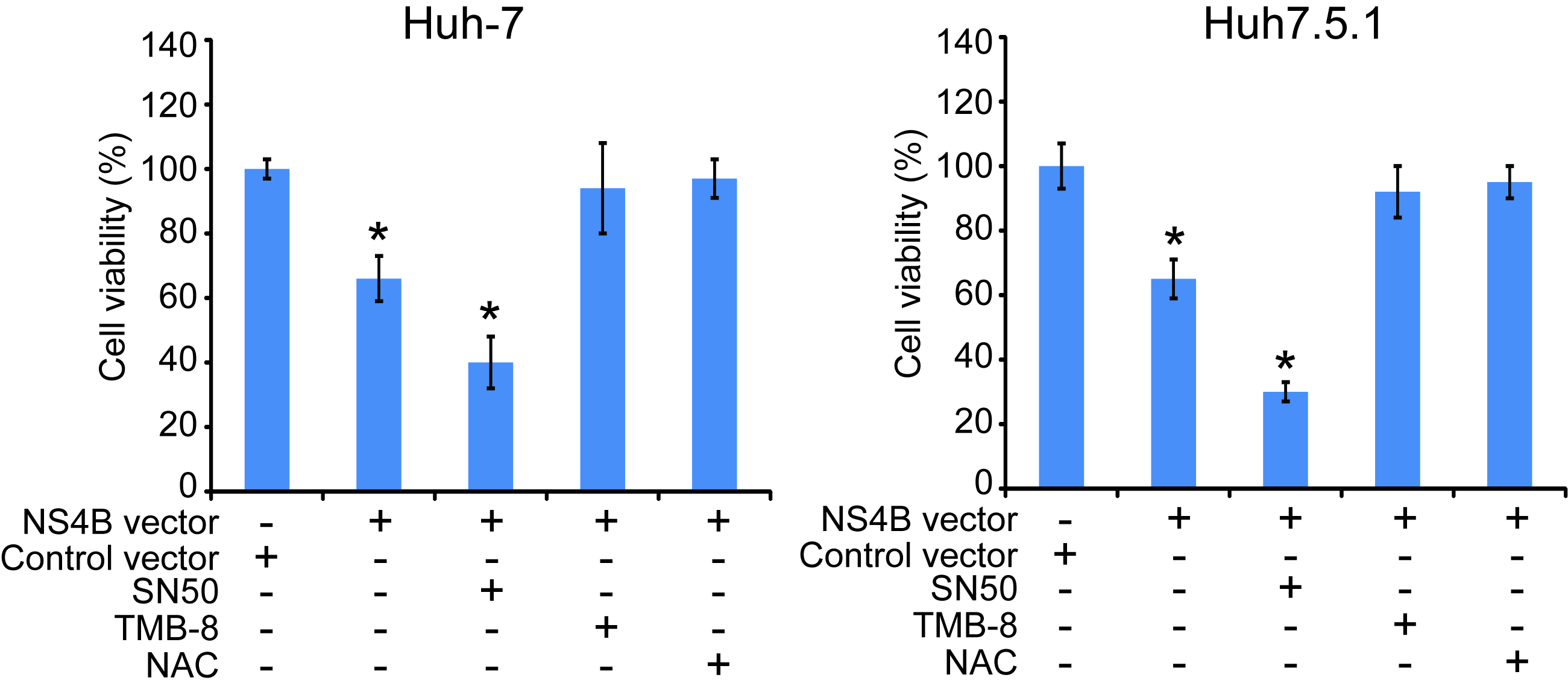

Supplement: S2 Fig — Huh-7 (A) or Huh7.5.1 (B) cells in 24-well plates were transfected with 0.4 μg pcDNA3.1(−)NS4B or 0.4 μg pcDNA3.1 (−), and treated with SN50 (40 μM) for 4 h, TMB-8 (100 μM) for 4 h, and NAC (30 mM) for 8 h as indicated. At 48 h posttransfection, cell viability was assessed using Cell Titre-Glo assay. Values are means ± SD (n = 3). * P < 0.05. (TIF) [file pone.0123190.s002.tif]

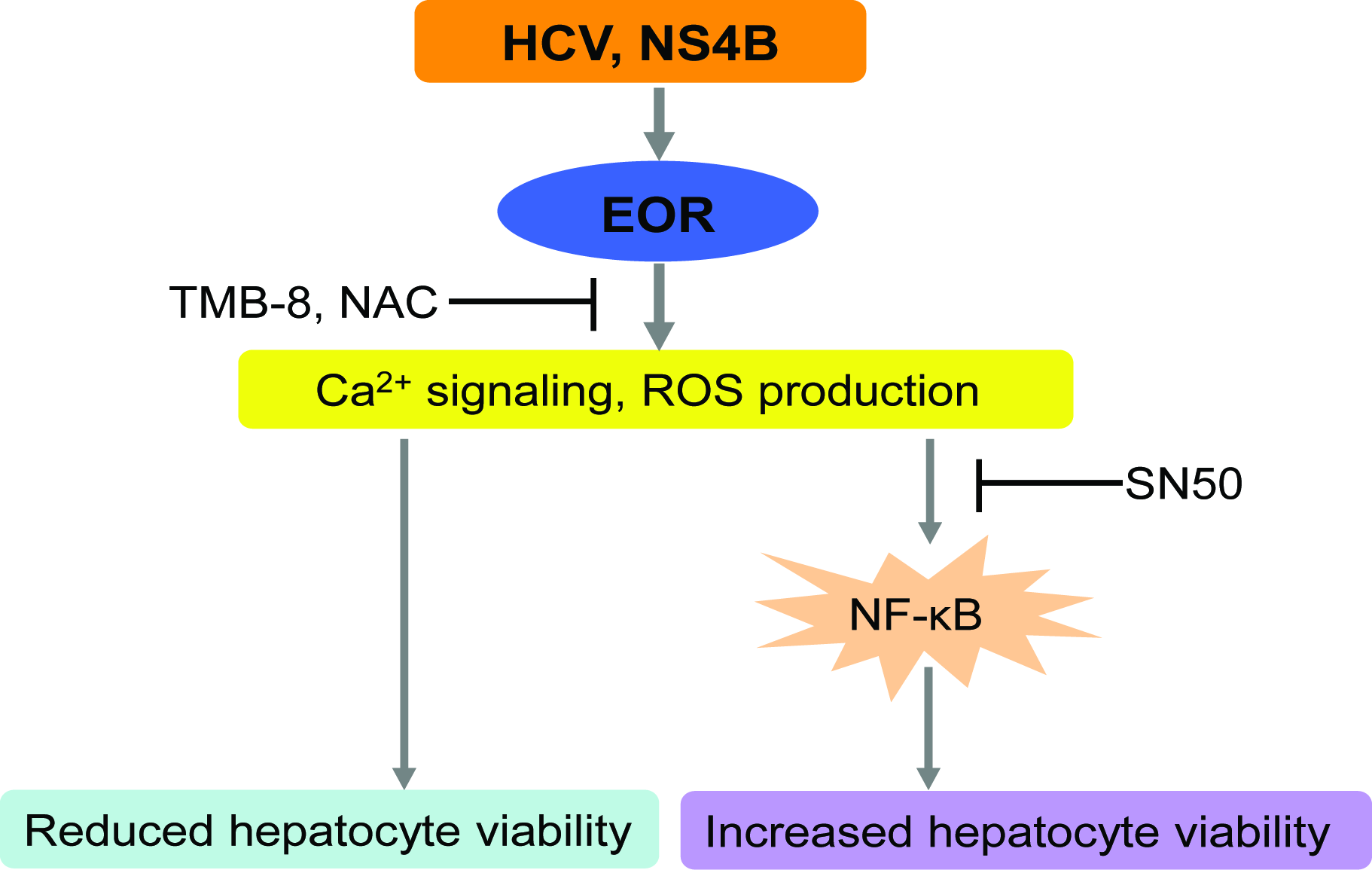

Supplement: S3 Fig — HCV and its protein NS4B induce the EOR-Ca2+-ROS pathway. Transient expression of NS4B and HCV infection induced cell death via Ca2+ signaling and ROS. Persistent expression of NS4B promoted human hepatocyte viability by Ca2+-ROS-activated NF-κB. SN50 specifically inhibits NF-κB, while TMB-8 and NAC specifically inhibit both EOR-Ca2+-ROS and EOR-Ca2+-ROS-NF-κB. (TIF) [file pone.0123190.s003.tif]

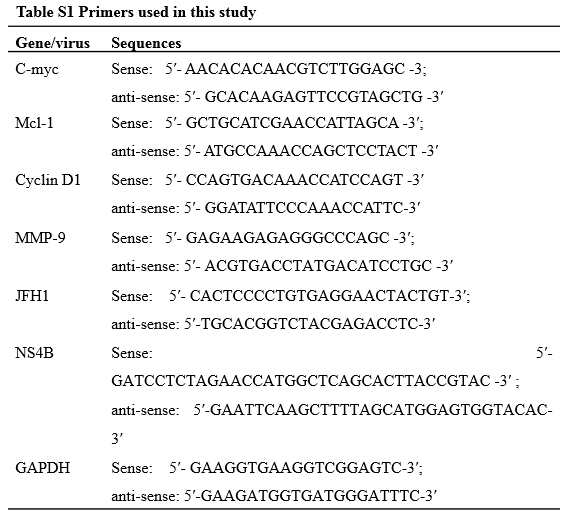

Supplement: S1 Table — (DOC) [file pone.0123190.s004.doc]
